# A Challenging Case of a Compound Calcaneum Fracture With Lateral Subtalar Dislocation Managed by Primary Subtalar Fusion: A Case Report

**DOI:** 10.7759/cureus.97032

**Published:** 2025-11-17

**Authors:** Saurabh Sah, Mulagondla Harshavardhan Reddy, Priyanshu, Ankush Mohabey, Deepanjan Das, Pratik Kotangale

**Affiliations:** 1 Orthopaedics, All India Institute of Medical Sciences, Nagpur, Nagpur, IND

**Keywords:** calcaneus fracture, lateral subtalar dislocation, post-traumatic arthritis, subtalar arthrodesis, subtalar fusion

## Abstract

Calcaneal fracture dislocations are relatively uncommon. Locked fracture dislocations of the calcaneus are particularly rare and often missed during initial evaluation due to subtle radiographic signs. As a result, many patients are initially treated symptomatically with rest and analgesics. Persistent pain and impaired function usually prompt further outpatient evaluation, leading to delayed diagnosis. This delay increases the risk of complications such as malunion and post-traumatic arthritis. Here, we present a case we managed, along with insights into the effective management of such complex injuries. A 14-year-old girl presented with persistent left foot pain, back pain, and inability to bear weight on the left foot for one week after a fall from a height. Examination revealed a 10 cm × 7 cm wound over the medial aspect of the foot, heel tenderness, and tenderness over the L4-L5 spinous processes. Imaging showed a calcaneal fracture with lateral subtalar dislocation and an L4-L5 compression fracture. After wound debridement and negative-pressure therapy, surgery was performed using a lateral extensile approach. Primary subtalar fusion with three 4.5 mm screws and K-wire stabilization of the talonavicular joint was performed. At six months, radiographs confirmed fusion, and the patient returned to normal, pain-free function. Primary subtalar fusion can serve as an effective treatment option in cases of open calcaneal fractures associated with subtalar dislocation, particularly when articular cartilage viability is compromised and joint preservation is not feasible. By addressing instability and preventing long-term pain from post-traumatic arthritis, fusion provides a stable and functional foot. However, because evidence is limited, further research with larger patient cohorts and long-term follow-up is essential to establish clear indications, functional outcomes, and potential complications. This will help define the precise role of primary subtalar arthrodesis in managing these complex injuries.

## Introduction

Calcaneal fracture-dislocations are relatively uncommon, comprising fewer than 2% of all foot and ankle fractures, with intra-articular ones accounting for about two-thirds of cases [1]. Calcaneum fracture with subtalar joint dislocation is a relatively rare injury, often the result of a high-energy trauma. Subtalar joint dislocation is classified into four types, namely, anterior, posterior, medial, and lateral, of which lateral dislocation is the rarest. Locked fracture-dislocations of the calcaneus are rare, and many of these injuries often go unrecognized or are misdiagnosed during the initial assessment. Due to the subtle signs present on plain radiographs, patients are frequently overlooked in terms of diagnosis and are typically managed with analgesics and rest. Patients often present to the outpatient department due to persistent pain and limited function, leading to further evaluation and eventual diagnosis. This delay in treatment can increase the risk of developing arthritic joints or malunion. Here, we present a case that we managed, along with our insights on the management of such injuries.

## Case presentation

**Figure 1:**
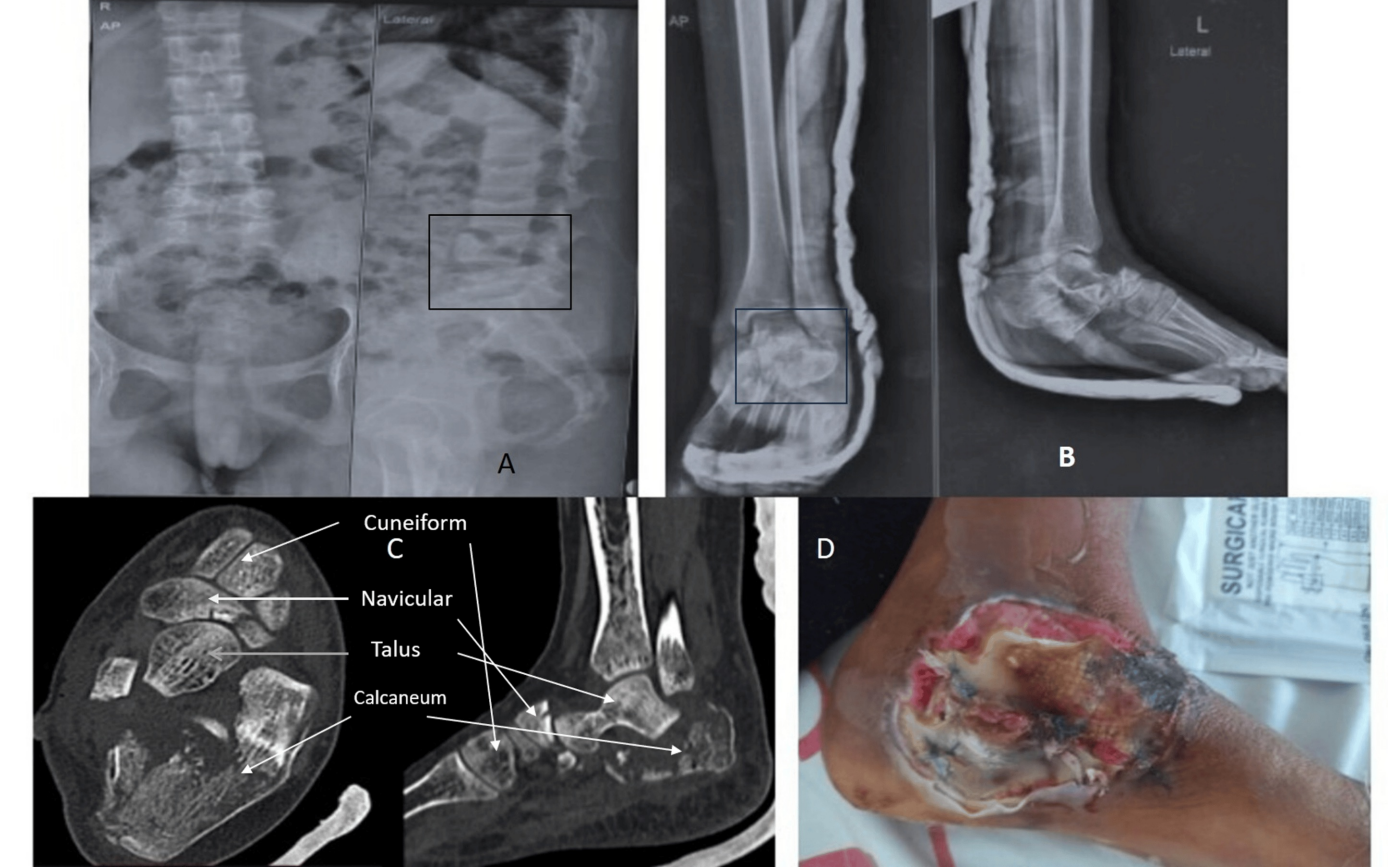
Preoperative scans and wound status. (a) Anteroposterior and lateral radiograph of the lumbar spine showing L4 and L5 wedge compression fracture. (B) Anteroposterior and lateral radiograph of the ankle with foot showing subtalar fracture-dislocation. (C) Cut section of foot CT depicting the pattern of the fracture. (D) Circular wound on the medial aspect of the foot, exhibiting slough and necrotic tissue.

A 14-year-old girl presented with persistent left foot pain, back pain, and inability to bear weight on the left foot for one week after a fall from a height. Initially evaluated by a local practitioner, her radiographs were read as normal, and she was advised to bear weight as tolerated. Due to ongoing symptoms, she sought further care at our hospital, where examination revealed a 10 × 7 cm wound with slough on the medial foot, swelling and tenderness over the heel, and tenderness at the lower lumbar spine. Plain radiographs showed a calcaneal fracture (Sanders classification type 3) with lateral subtalar dislocation and compression fractures at L4 and L5. A CT scan confirmed an intra-articular calcaneal fracture with anterior process involvement with lateral subtalar dislocation (Figure 1).

**Figure 2:**
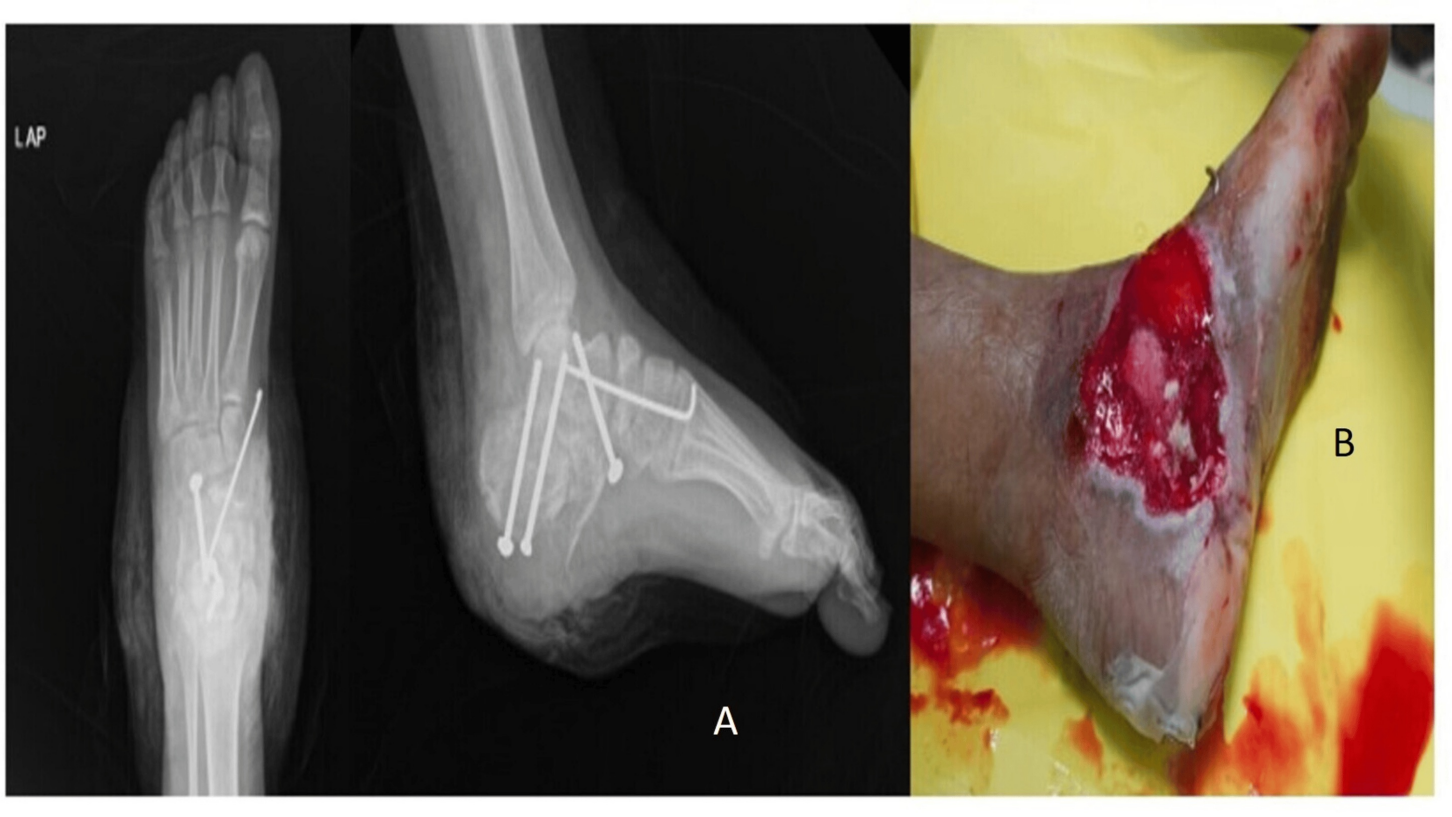
Postoperative radiograph and post-vacuum-assisted closure wound. (A) Postoperative anteroposterior and lateral view of foot radiograph showing fixation with screws and dislocation reduced by K-wire. (B) Wound post-debridement and vacuum-assisted closure application for seven days showing healthy granulation tissue.

Surgery was delayed to manage the wound, which was treated with debridement and negative-pressure therapy. The patient was kept on intravenous antibiotics, which included ceftriaxone, metronidazole, and amikacin. Metronidazole was stopped after five days of presentation, and amikacin was stopped after seven days of presentation, after confirming no active infection by the absence of purulent or serous discharge and downgrading levels of C-reactive protein values every alternate day. Intraoperative cultures were negative for organism growth. Two weeks later, operative intervention was performed using a lateral extensile approach. The reduction of the subtalar joint was done and fused using three 4.5 mm cannulated screws and was stabilized with a K-wire (Figure 2). The cartilage was curetted out using a bone curette.

**Figure 3:**
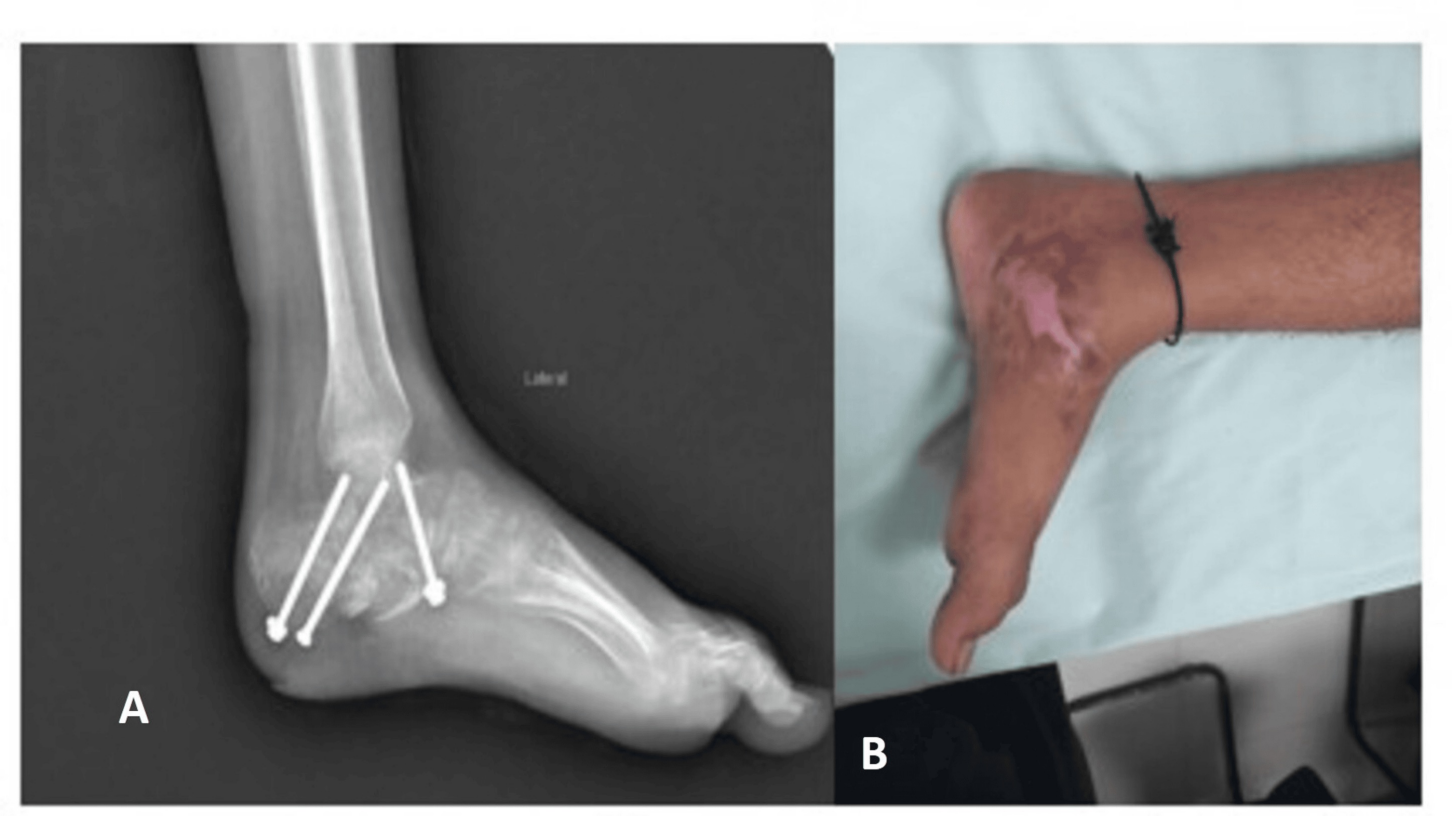
Follow-up radiograph and healed scar. (A) Anteroposterior and lateral three-month follow-up radiograph of the foot showing screws in position and evidence of healing. (B) Wound healed by secondary intention by granulation tissue.

Postoperatively, the patient was kept non-weight-bearing in a below-knee splint. At two weeks, the surgical site had healed well, and the compound wound over the medial aspect was granulating, following which sutures were removed. By three months, weight-bearing and ankle mobility exercises were initiated (Figure 3). At six months, radiographs confirmed solid fusion, and the patient had regained full function and resumed normal activities without pain or limitations. Visual Analog Scale scores improved from 8 on presentation to 1 after three months, and the Foot Function Index improved from 136 to 27. Table 1 presents a timeline of the patient presentation and management.

**Table 1:** Timeline of events.

Time from presentation	Event
After 1 week of injury	Presented to our outpatient department
2nd day	Debridement with negative-pressure therapy
2nd week	Subtalar fusion
4th week	Suture removal
6th week	Ankle pumps and range of motion
8th week	Partial weight-bearing mobilization
12th week	Full weight-bearing mobilization

## Discussion

Subtalar dislocation involves simultaneous displacement of the talonavicular and talocalcaneal joints, with preservation of the tibiotalar and calcaneocuboid joints [2]. It typically results from high axial force on an inverted foot, causing ligament disruption and calcaneal fragment displacement, which complicates closed reduction [3]. These injuries are often missed on initial radiographs, increasing the risk of long-term complications such as arthritis and avascular necrosis. Lateral dislocations may involve peroneal tendon instability due to anterior tendon displacement and avulsion of the superior peroneal retinaculum [4].

The decision to perform primary fusion rather than initial open reduction and internal fixation (ORIF) is based on the severity of the articular surface damage, chondral loss, and the associated compound injury. While ORIF remains a standard treatment for many calcaneal fractures, primary fusion can be a viable alternative in cases where joint preservation is unlikely to yield satisfactory outcomes [5]. Subtalar fusion in such cases offers the best chance for long-term functional stability, pain relief, and prevention of degenerative joint disease [6]. Subtalar fusion was favored in this case as a single-stage procedure, reducing the need for multiple surgeries and lowering the overall treatment burden. It also allows for a shorter rehabilitation period while providing satisfactory functional outcomes [7]. The fusion provides stability to the joint, alleviates pain, and helps restore the ability to perform daily activities, which is particularly important in the context of a compound injury and the patient’s overall condition.

A suggested advantage of subtalar fusion and similar techniques is the improvement in hindfoot alignment and joint congruity, which is achieved without causing extensive disruption to the surrounding soft tissues [8]. Early recognition and prompt management of such injuries are critical to prevent complications such as a widened hind foot, minimize soft tissue damage, and delay the onset of degenerative changes in the joint [8]. This is particularly beneficial in high-risk populations, where minimizing soft tissue damage is crucial to reducing the likelihood of wound complications, delayed healing, and infection [9].

The outcomes observed in this case demonstrate the potential benefits of primary subtalar fusion. While primary subtalar fusion offers immediate stability and potential benefits for open calcaneum fractures with lateral subtalar dislocation, it may result in limited hindfoot motion compared to traditional ORIF techniques, necessitating careful consideration between early functional recovery and long-term joint mobility [10]. The double density sign on lateral X-rays is a key diagnostic indicator of these complex injuries [1].

Long-term follow-up is crucial to assess the durability of the fusion and monitor for potential complications such as adjacent joint arthritis. Additionally, comparing the outcomes of this approach with those of traditional management strategies in similar injury patterns would provide valuable insights for future treatment protocols.

## Conclusions

In the case of closed subtalar fracture dislocation, ideal treatment would have been reduction of the subtalar joint and fixation of the calcaneum fracture. However, faced with a compound injury where the viability of the articular cartilage was compromised, we opted to perform a primary subtalar arthrodesis. The functional outcome in our case was good. Primary subtalar fusion can be a good treatment option for open calcaneum fractures with subtalar dislocation. Further research and long-term follow-up studies are needed to establish the optimal indications and long-term outcomes of this approach in managing these complex injuries.
